# Pharmacists as Interprofessional Collaborators and Leaders through Clinical Pathways

**DOI:** 10.3390/pharmacy6010024

**Published:** 2018-03-16

**Authors:** Sherine Ismail, Mohamed Osman, Rayf Abulezz, Hani Alhamdan, K. H. Mujtaba Quadri

**Affiliations:** 1 King Abdullah International Medical Research Center, King Saud bin Abdulaziz University for Health Sciences, Pharmaceutical Care Department, King Khalid Hospital, Ministry of National Guard Health Affairs, Jeddah 21423, Saudi Arabia; hamdanhs@ngha.med.sa; 2 Trillium Health Partners, Credit Valley Hospital, Mississauga, ON L5M 2N1, Canada; mohd67osman@hotmail.com; 3 King Abdullah International Medical Research Center, King Saud bin Abdulaziz University for Health Sciences, Pharmaceutical Care Department, Prince Mohammed Bin Abdulaziz Hospital, Ministry of National Guard Health Affairs, Madinah 41511, Saudi Arabia; Abualezzrs@ngha.med.sa; 4 National University of Medical Sciences, The Mall, Rawalpindi 44000, Pakistan; deanresearch@numspak.edu.pk

**Keywords:** clinical pathways, pharmacists, clinical pharmacists, interprofessional collaboration, integrated care and patient-centered outcomes

## Abstract

Pharmacists possess pivotal competencies and expertise in developing clinical pathways (CPs). We present a tertiary care facility experience of pharmacists vis-a-vis interprofessional collaboration for designing and implementing CPs. We participated in the development of CPs as leading members of a collaborative team of healthcare professionals. We reviewed literature, aligning it with hospital formulary and institutional standards, and participated in weekly team meetings for six months. Several tools and services were adapted to guide prescribing and standardization of care through time-bound order sets. Fifteen CPs leading to admissions in medical wards were developed and integrated into Computerized Prescriber Order Entry (CPOE) sets. Tools and services included (1) reporting of creatinine clearance to guide optimum dosing; (2) advisory flags for dosing and infusion rates; (3) piloting of medication reconciliation and counseling services before discharge were initiated; (4) Arabic drug leaflets were designed to educate patients; and (5) five CPs were included in pragmatic randomized control trials with a clinical pharmacist as co-investigator. Clinical pharmacists conducted continuous orientation to various healthcare professionals throughout the process. CPs provide unique opportunities for establishing and evaluating patient-centered pharmaceutical services and allow clinical pharmacists to demonstrate interprofessional leadership in collaboration with multidisciplinary teams.

## 1. Introduction

Pharmacists are pharmacotherapy experts, and possess pivotal skills which qualify them for playing active roles in the process of designing and application of clinical pathways (CPs) [1,2]. Literature has consistently reported that CPs can be differentiated from guidelines [3,4] in that CPs are time-bound patient care plans which aim to improve the quality of patient-care and optimize utilization of institutional resources [5,6]. 

The introduction of CPs in medical institutions was a result of the paradigm shift in the healthcare system from the quantitative aspect to focus on the quality of care and target patient-centered outcomes [7]. Additionally, the designing of CPs requires the presence of a dedicated team of multidisciplinary healthcare professionals who work collaboratively to develop evidence-based and patient-oriented pathways for high-volume, high-risk, and/or high-cost diagnoses [5,8]. 

A systematic review composed of twenty-seven studies that included 11,398 participants reported that CPs reduced in-hospital complications and increased the rate of documentation of the staff, but authors were not able to poll results for length of stay (LOS) [9]. Another systematic review has demonstrated that CPs had a significant decline in LOS in 12 out of 16 studies analyzed, with a weighted mean difference of −2.5 days for CPs vs. −0.8 days for the standard of care, and 4 out of 6 studies showed a decline in the costs for CPs [10]. However, the majority of CPs studied were in the surgical setting, reported high heterogeneity for LOS, and the effectiveness of CPs remained uncertain [10]. Likewise, the Department of Medicine in our Joint Commission International (JCI) accredited institution aimed to design CPs and to answer the question of the utility of CPs to improve the flow of patients and medical care across multiple medical diagnoses. 

The American College of Clinical Pharmacists (ACCP) encourages pharmacists to embrace CPs as opportunities to deliver multifaceted pharmaceutical care [11]. Additionally, the American Society of Health-System Pharmacists (ASHP) provides clear guidelines on the responsibilities of pharmacists in the development, implementation, and assessment of CPs [12]. Furthermore, both the ACCP and ASHP have identified CPs as tools for pharmacists to provide cost-effective patient care plans, integrate pharmaceutical services, institutional culture, and partake leadership position in the development and implementation of the process [12,13]. 

To date, the literature describing a practice-based prototype of how pharmacists were engaged in the designing and the application of CPs is scarce. Therefore, we aim to describe our experience in the development, implementation, and assessment of CPs as a model of interprofessional collaboration in improving patient-centered outcomes.

## 2. Materials and Methods 

### 2.1. Development of Pathway Team and Pharmacy Team

The Department of Medicine at King Abdulaziz Medical City, Jeddah, Saudi Arabia invited various healthcare professionals in 2011 to formulate a team composed of physicians, nurses, pharmacists, quality specialists, dietitians, social workers, discharge planning, primary health care physicians, and patient educators. The objective of the interprofessional collaborative team was to provide a holistic approach in designing evidence-based and patient-centered pathways. CPs were defined as time-bound plans to deliver patient care from the admission till the discharge day by all healthcare professionals for specific medical diagnoses. 

In response to the invitation, Pharmaceutical Care Department designated a team of pharmacists to provide strategic planning for the participation of the pharmacy and collaboration with the pathway team. The pharmacy team included internal medicine clinical pharmacists who are Board Certified Pharmacotherapy Specialists, inpatient, IV admixture team, and clinical pharmacy supervisors. Additionally, a clinical pharmacist was assigned as a pharmacy coordinator to harmonize the perspectives of the pharmacy team in synchrony with the vision of the pathway team. 

### 2.2. Perspectives of the Pharmacy Team

The pharmacy team set up the following goals and perspectives of pharmaceutical care services as detailed in Table 1.

**Figure 1:**
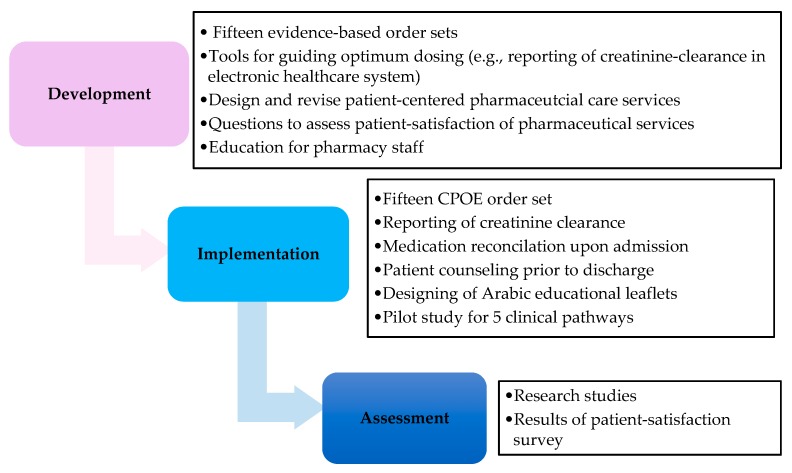
Layout of the roles of pharmacists as interprofessional collaborators in CPs.

A summary of our interprofessional collaboration based on ACCP and ASHP standards for the roles of pharmacists in the designing and the application of CPs [12,13,14] is demonstrated in Figure 1.

### 2.3. The Development Phase

#### 2.3.1. Order Sets

The pathway team targeted the fifteen most frequent admitting diagnoses in medical wards for designing CPs. The development phase was carried out over a period of 6 months. The coordinating clinical pharmacist conducted an evidence-based literature review, designed order sets for each medical diagnosis, and participated in discussions and appraisal of evidence with members of the pathway team and specialty physicians on a regular weekly basis. Furthermore, order sets for each CP were reviewed by the pharmacy team for feasibility of implementation and suggested changes were communicated back to pathway team through the coordinating clinical pharmacist. The order sets included cost-effective therapeutic plans on a daily basis during the hospital stay for each medical diagnosis, and were designed to comply with safety measures for prescribing according to the Institute of Safe Medication Practice for standard order sets [15].

#### 2.3.2. Patient-Centered Pharmaceutical Care Services

We aimed to conduct medication reconciliation by pharmacists in collaboration with physicians based on Best Possible Medication History (BPMH) [16], to optimize patient safety upon transition of care [17]. Additionally, we redesigned our counseling team to provide patient counseling based on Indian Health Services counseling technique [18], and ASHP standards [19]. Furthermore, we designed educational leaflets in the Arabic language to enhance the education of patients during counseling before discharge. Subsequently, we trained our pharmacy staff working in the inpatient and to take-home medications using role-playing sessions to standardize their performance and provide consistent practical experience for patient-centered services. Both services of medication reconciliation and counseling were carried out during working days only, and involved designated pharmacy personnel.

#### 2.3.3. Tools

We coordinated the integration of the reporting of creatinine clearance in the electronic healthcare system after several meetings with Nephrology team as the major stakeholders and informatics technology. We aimed to facilitate the assessment of kidney function to guide optimum drug dosing for renal patients along with order sets, which served as a clinical decision support system for healthcare providers [20,21]. In addition, we incorporated advisory flags in the order sets for maximum infusion rates and dosing for medications based on the therapeutic indications and special clinical situations for each CPs. Furthermore, we activated the documentation of the therapeutic interventions by pharmacists in the electronic medical records. Finally, detailed information based on the interview during counseling and medication reconciliation was documented to improve the communication process between pharmacists and other healthcare professionals, thus facilitating holistic patient care.

#### 2.3.4. Research Opportunities

The Department of Medicine aimed to conduct a study to assess the effectiveness of CPs through a Collaborative Healthcare Approach in Monitoring Patient-centered outcomes through Pathways (CHAMP-Path) studies. These are pragmatic, randomized, single-blinded studies comparing five CPs vs usual care to reduce the length of stay and improve patient-centered outcomes. Clinical pharmacists with research certification were invited to participate in the study as leading co-investigators to revise and submit the proposal of the study to the Institutional Review Board (IRB) for approval. Details for the method of the CHAMP-Path study have been reported [22]. The pharmacy was responsible for allocation of the study participants. Additionally, the study included a survey to assess the level of patient satisfaction with the services provided by all healthcare professionals. We designed five questions as a part of the survey to assess the perceptions of patients towards pharmaceutical care services, which are included in Table 2.

##### Statistical Analyses

Survey responses were presented as proportions and 95% Confidence interval. STATA 2014 (StataCorp LLC, College Station, TX, USA) was used for statistical analysis. 

##### Ethics

The CHAMP-Path study received IRB approval by King Abdullah International Medical Research Center ((RC 10/134/J) in October 2011. Informed consents were obtained for eligible participants.

## 3. Results

### 3.1. Implementation

#### 3.1.1. Order Sets

Fifteen (100%) CPs were developed in collaboration with the pathway team, including acute kidney injury, venous thromboembolism, community-acquired pneumonia, asthma, adult left ventricular heart failure, chronic kidney injury, upper gastro-intestinal bleeding, ischemic stroke, hepatic encephalopathy, generalized seizures, palliative care, acute coronary syndrome, meningitis, diabetic ketoacidosis, and hyperosmolar hyperglycemia. 

**Figure 2:**
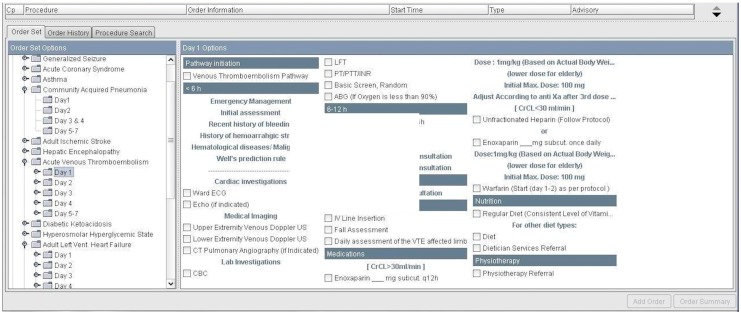
An example of Computerized Order Prescriber Entry of day one for venous thromboembolism pathway.

The order sets of the therapeutic regimens for all 15 CPs were integrated into CPOE over a period of three months through collaboration with pathway team and information technology department. Subsequently, CPOE order sets were reviewed by the clinical pharmacist coordinator and the chair of pathway team to ascertain the accuracy and validity for use in direct patient care. Figure 2 is a screenshot of day one for an electronic CPOE order sets for venous thromboembolism.

A pilot study of five clinical pathways started for 6 months in March 2012. We worked with physicians on updating the therapeutic components of CPs during the implementation period based on recent guidelines or new studies. Additionally, we maintained effective communication strategies with CPs team, which facilitated the integration of these therapeutic updates into CPOE order sets promptly.

#### 3.1.2. Patient-Centered Pharmaceutical Care Services

Medication reconciliation by the pharmacist within 24 h of admission started as a pilot phase. The pharmacists provided education for patients and utilized educational leaflets to improve patient’s knowledge about their medications. Pharmacists communicated with the physicians for possible necessary changes upon order verification, and documented their therapeutic interventions during the patient interview in the electronic healthcare system.

#### 3.1.3. Tools

Creatinine clearance estimation was reported in the electronic system as well as all cautionary and advisory flags developed in the order sets.

#### 3.1.4. Research 

The coordinating clinical pharmacist worked with the research team as a co-investigator. The study received IRB approval, and five out of fifteen (33%) of CPs were studied in CHAMP-Path study. The five CPs were acute kidney injury, venous thromboembolism, asthma, community acquired pneumonia, and heart failure. Emergency Pharmacy was responsible for the allocation of study participants as per the randomization scheme. The coordinating clinical pharmacist with the CHAMP-Path team participated in the presentation of the study updates on an annual basis at King Abdullah International Research forums from 2012 to 2015. Furthermore, the study method and collaboration of pharmacy with the multidisciplinary team were presented at other international research forums, such as The Principles and Practice of Clinical Research course in Sao Paulo, Brazil in 2011 offered by Harvard T H Chan School of Public Health and the Global Conference of the American College of Clinical Pharmacy, CA, United States in 2015. Finally, to date, two papers reporting CHAMP-Path studies were published with the coordinating clinical pharmacist as the leading author or co-author [22,23]. In the acute kidney injury study, the primary outcome of median length of stay (LOS) was 4.96 days (interquartile range of 6.57) for the pathway care compared to 4.8 days (interquartile range of 6.84 days) for the usual care (*p* = 0.8). Secondary outcomes of 30-day readmission and in-hospital mortality were also not statistically different [23]. Preliminary findings of unpublished data demonstrated that heart failure and venous thromboembolism showed a significant reduction in primary outcome of LOS and further data analysis for the findings of the studies is ongoing.

### 3.2. Assessment

#### 3.2.1. Pilot Study for Validation of Patient-Satisfaction Survey

A pilot study of 20 participants was conducted to assess the validity of CHAMP-Path patient-satisfaction survey. We present the results focusing on the questions related to the pharmacy section. Forty-five percent (9/20) of respondents requested clarifications when asked if pharmacy reviewed their home medication within 24 h of admission, and 30% (6/17) of respondents did not understand the question on the medication reconciliation process. Almost one-quarter of respondents, 26.7% (4/15), asked for clarifications about receiving counseling before discharge, 20% (3/15) had some questions regarding the overall performance of pharmaceutical services, and only 13.3% (2/15) inquired about the question pertaining to their understanding of information during the counseling process. 

The Cronbach’s alpha for internal consistency was 0.39 for pharmaceutical care questions, which were attributed to long questions, word phrasing, and to the fact that medication reconciliation services by pharmacists were not activated at the pilot phase. Subsequently, the survey questions and responses for pharmaceutical services were revised, and the final survey questions for pharmaceutical services are presented in Table 3.

#### 3.2.2. Patient-Satisfaction Survey 

We had 338 patients who were enrolled in the CHAMP-Path study from 2012 to 2016, of which 182 (53.85%) completed the patient-satisfaction survey. Some patients had missing responses for the questions related to pharmaceutical services. The results of the patient-satisfaction survey related to pharmaceutical services are presented in Table 4.

#### 3.2.3. Continuous Education

Clinical pharmacy coordinator presented regular orientation sessions for pharmacy staff on their roles and duties during the implementation process of CPs. Furthermore, we actively participated in the pathway workshops to enhance the awareness of various healthcare professionals on the process of pathway development and strategies for integration into CPs. Additionally, the clinical pharmacy coordinator collaborated with pathway team to provide regular annual orientations to the medical residents on the use of CPOE order sets and the study-related logistics, such as screening and enrollment.

## 4. Discussion

Our interprofessional collaboration with the multidisciplinary team of healthcare professionals has paved the path for various opportunities to provide patient-centered pharmacist care through multifaceted interventions. As we did not have an explicit practice model, we strove to follow the standards of ACCP, ASHP, ISMP, and international guidance on the role of the pharmacist in designing CPs. The results of the patient-satisfaction survey demonstrated that counseling services were useful in improving the perception of 86.9% of respondents about their medications, which is an essential step for adherence and reducing hospital readmission. A systematic review demonstrated that patient counseling reduced morbidities, mortalities, and enhanced interprofessional collaboration [24]. Although 71.7% of respondents to the survey in our study received medication reconciliation, only 56.6% rated the service as good or very good. The low rate of satisfaction demonstrates an area for improvement in our setting. However, a study conducted at our hospital in 2012–2013 comparing medication reconciliation by pharmacist vs physician, and included >50% of medical patients, demonstrated a significant difference in the number of discrepancy medications identified by the pharmacist vs physicians [25]. These findings warrant the need to revisit the consistency and sustainability of the quality of medication reconciliation services by pharmacists in our setting, which have demonstrated their effectiveness in optimizing patient-care and medication safety [26].

Our collaborative experience had several limitations and challenges: (1) Although we worked to develop fifteen CPs, we were able to assess the outcomes of only five of these CPs, which were included in the CHAMP-Path study due to obstacles in randomizing physicians into teams for other subspecialties in a pragmatic randomized-controlled trial; (2) We were not able to monitor for the adherence to the use of CPOE order set, due to technical difficulties as well as the nature of the pragmatic design [27,28], which allows physicians to deviate from CPs to meet individual patients’ need; (3) We had periods of inconsistent pharmaceutical care services, such as medication counseling and reconciliation during weekend and holidays due to shortage of staffing, which interrupts the continuity of care and undermines the effect of these services on patient-centered outcomes. Furthermore, we did not assess therapeutic interventions by pharmacists and their effect on patient care, due to technical issues in retrieving these therapeutic interventions for auditing purposes. 

We have identified several strengths in our experience in the development and implementation of CPs: first, it presents a unique model for pragmatic interprofessional collaboration with various multidisciplinary teams aiming to improve patient-centered outcomes. Interprofessional collaboration is endorsed by the Institute of Medicine for incorporation in the educational curriculum to empower the future generation of practitioners with necessary skills and competencies [29]. Second, CPs facilitated piloting, launching of many patient-centered pharmaceutical care services, and engaging pharmacists in clinical research. Additionally, it delivered key messages on areas for improvement and demonstrated the flexibility of pharmacists to changes to achieve the desired strategic goals of the institution. Third, it offered leadership opportunities for clinical pharmacists, as stakeholders of therapeutics in the organization, to provide safe and cost-effective medication regimens [7]. Future studies assessing clinical pathways should describe further practice models for interprofessional collaboration for pharmacists and pharmaceutical services targeting improved clinical outcomes.

## 5. Conclusions

Clinical pathways provide unique opportunities for establishing and evaluating patient-centered pharmaceutical services, and allowing pharmacists to demonstrate interprofessional leadership skills in collaboration with multidisciplinary teams.

## Figures and Tables

**Table 1:** Goals and Perspectives of the Pharmacy team.

Collaborate with multidisciplinary pathway team to provide evidence-based, patient-centered therapeutic regimens in the form of order sets within clinical pathways (CPs) to achieve the goals of the Department of Medicine and the institution. Align CPs with formulary decisions by the Pharmacy and Therapeutics Committee: use of formulary medications, facilitate the adherence to the approved restricted medications, integrate institutional drug use policies and JCI measures to maximize patient safety and seek for optimum use of therapeutic regimens through CPs. Pilot pharmaceutical services such as medication reconciliation within 24 h of admission, patient counseling before discharge and documentation of therapeutic interventions by pharmacists. Design effective tools to implement these perspectives such as the integration of order sets into CPOE to optimize the use of standardized cost-effective and safe therapeutic regimens. Communicate with healthcare professionals effectively to enhance the implementation of these tools. Identify opportunities within the pathway team to optimize the cost-effective use of medications. Provide continuous education to pharmacy staff in CPs and other healthcare professionals on the strategies for employing CPs. Sustain a consistent performance for pharmaceutical activities and services in collaboration with pathway team.

**Table 2:** Questions related to pharmaceutical care services in the pilot phase of CHAMP-Path patient-satisfaction survey.

Pharmaceutical Care (الرعاية الصيدلانية)	Responses				
1 Did the Pharmacist review your home medication within 24 h of admission? هل راجع الصيدلي أدويتك الخاصة بك التى تتناولها بالمنزل خلال 24 ساعة من تنويمك؟	Yes ( نعم ) □		No ( لا ) □		
2 Did you receive counseling by the Pharmacist on your medications before discharge? هل حصلت على معلومات خاصة بادويتك من الصيدلي قبل خروجك من المستشفى؟	Yes ( نعم ) □		No ( لا ) □		
	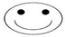	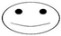	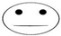	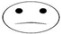	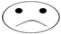
	Excellent	Very Good	Good	Poor	Unsatisfactory
	ممتا ز	جيد جدا	جيد	ضعيف	غير مقبول
3 How would you describe the process of reviewing your home medication with the Pharmacist upon admission? كيف تصف طريقة مراجعة أدويتك الخاصة مع الصيدلي عند التنويم؟	□	□	□	□	□
4 How would you best describe your level of understanding about your medications based on the educational information you received from your pharmacist before discharge? كيف تصف مستوى فهمك للأدوية الخاصة بك حسب التعليمات التي تلقيتها من الصيدلي قبل خروجك من المستشفى؟	□	□	□	□	□
5 How would you best describe the overall performance of the pharmaceutical services provided during your stay in hospital? كيف تصف الأداء العام للخدمات الصيدلانية المقدمة خلال إقامتك في المستشفى؟	□	□	□	□	□

**Table 3:** Questions related to pharmaceutical care services in the CHAMP-Path patient satisfaction survey.

	Pharmaceutical Care						الرعاية الصيدلانية	
1	Did the Pharmacist review your home medications?	Yes		□	نعم		هل راجع الصيدلي أدويتك التى تتناولها بالمنزل؟	١ـ
		No		□	لا			
		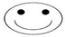	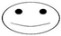	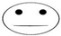	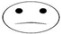	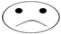		
		جيد جدا	جيدا	محايد	ضعيف	ضعيف جدا		
		Very Good	Good	Neutral	Poor	Very Poor		
2	How would you rate the process of reviewing your home medication with the Pharmacist upon admission?	□	□	□	□	□	ما هو تقييمك لطريقة مراجعة أدويتك مع الصيدلي عند دخولك في المستشفى؟	٢ـ
		Did not Review		□	لم يراجع أدويتي			
3	Has the pharmacist counseled you on the medications, which you will be taking home with you?	Yes		□	نعم		هل نصحك الصيدلي عن الأدوية التي ستأخذ معك إلى المنزل؟	٣ـ
		No		□	لا			
		Not Applicable		□	لا ينطبق			
		(Discharged after-hours)						
		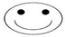	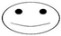	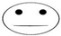	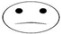	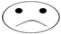		٤ـ
		فهمت تماما	فهمت كثيرا	فهمت نوعا ما	فهمت قليلا	لم أفهم		
		Completely understood	Understood a lot	Understood somewhat	Understood a little	Did not understand		
4	How would you rate your level of understanding about your medications based on the educational information you received from your pharmacist before discharge?	□	□	□	□	□	ما هو تقييمك لمستوى فهمك للأدوية الخاصة بك حسب التعليمات التي تلقيتها من الصيدلي قبل خروجك من المستشفى؟	-٥
		No Information		□	لم أتلق أي معلومات			
		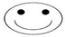	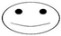	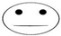	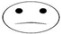	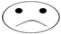		
		جيد جيد جدا	جيد ا	محايد	ضعيف	ضعيف جدا		
		Very Good	Good	Neutral	Poor	Very Poor		
5	How would you rate the overall performance of the pharmaceutical services provided during your stay in hospital?	□	□	□	□	□	ما هو تقييمك عموما للخدمات الصيدلية المقدمة خلال إقامتك في المستشفى؟	٦ـ

**Table 4:** Results of CHAMP-Path patient-satisfaction survey related to pharmaceutical care services.

	Questions	Responses	Proportions n/N (%)	95% Confidence Intervals
**Medication Reconciliation upon admission**				
1	Received medication reconciliation by pharmacist	Yes	119/166 (71.7)	64.8–78.6
2	Evaluation of Medication reconciliation by pharmacist	Did not review	49/159 (30.8)	23.6–38.0
		Poor ^a^	20/159 (12.6)	7.4–17.8
		Good ^b^	90/159 (56.6)	48.9–64.3
**Patient counseling before discharge**				
3	Received counseling by pharmacist	Yes	102/147 (69.4)	62.0–76.8
		Not applicable ^c^	28/147 (19.0)	12.7–25.3
4	Level of understanding about medications based on counseling by pharmacist	No information provided	14/145 (9.7)	4.9–14.5
		Poor understanding ^d^	5/145 (3.4)	0.5–6.3
		Good understanding ^e^	126/145 (86.9)	81.4–92.4
**Overall performance of Pharmaceutical Services**				
5	Evaluation of overall performance of the pharmaceutical services provided	Poor ^a^	38/144 (26.4)	19.2–33.6
		Good ^b^	106/144 (73.6)	66.4–80.8

^a^ Poor: Poor is a collapsed category of very poor, poor and neutral; ^b^ Good: good is a collapsed category of good and very good; ^c^ Not applicable was due to discharge during the weekend or patient discharge after working hours for counseling pharmacist; ^d^ Poor understanding: is a collapsed category of did not understand and understood a little; ^e^ Good understanding: is a collapsed category of somewhat understand, understood a lot and understood completely.
